# Emotional eating in women with generalized anxiety disorder

**DOI:** 10.47626/2237-6089-2021-0399

**Published:** 2023-09-22

**Authors:** Natasha Kim de Oliveira da Fonseca, Marianna de Abreu Costa, Natan Pereira Gosmann, Roberta Dalle Molle, Francine Guimarães Gonçalves, Alice Cardozo Silva, Ylana Rodrigues, Patrícia Pelufo Silveira, Gisele Gus Manfro

**Affiliations:** 1 Programa de Pó́s-Graduação em Psiquiatria e Ciências do Comportamento Universidade Federal do Rio Grande do Sul Porto Alegre RS Brazil Programa de Pó́s-Graduação em Psiquiatria e Ciências do Comportamento, Universidade Federal do Rio Grande do Sul (UFRGS), Porto Alegre, RS, Brazil.; 2 Department of Psychiatry Faculty of Medicine McGill University Montreal Canada Department of Psychiatry, Faculty of Medicine, McGill University, Montreal, Canada.; 3 UFRGS Porto Alegre RS Brazil Curso de Graduação em Nutrição, UFRGS, Porto Alegre, RS, Brazil.; 4 Ludmer Centre for Neuroinformatics and Mental Health Douglas Mental Health University Institute Montreal Canada Ludmer Centre for Neuroinformatics and Mental Health, Douglas Mental Health University Institute, Montreal, Canada.

**Keywords:** Emotional eating, generalized anxiety disorder, emotional dysregulation, self-compassion

## Abstract

**Introduction:**

Individuals diagnosed with generalized anxiety disorder (GAD) seek pleasurable foods to avoid their negative emotional experiences. Ineffective regulation of negative emotions may be a risk factor for emotional eating (EE), leading to suffering, dysfunctional behaviors, and weight gain.

**Objectives:**

The aim of this study is to understand the relationship between emotional dysregulation and EE, investigating potential mediators such as the intensity of the worry, avoidance of internal experiences, mindfulness, and self-compassion in female patients with anxiety.

**Methods:**

In this cross-sectional study, participants from a randomized clinical trial diagnosed with GAD answered the following instruments at baseline: the Difficulties in Emotion Regulation Scale (DERS), the Three Factor Eating Questionnaire (TFEQ-R21), the Penn State Worry Questionnaire (PSWQ), the Action and Acceptance Questionnaire (AAQ), the Five Facet Mindfulness Questionnaire (FFMQ), and the Self-Compassion Scale (SCS). We estimated Pearson correlation coefficients and performed mediation analyses.

**Results:**

We evaluated 51 female individuals, 34 of whom completed all the questionnaires. Our data showed that EE was positively correlated with emotional dysregulation (r = 0.593; p < 0.001), worry trait (r = 0.402; p = 0.018), and avoidance of internal experiences (r = 0.565; p < 0.001), whereas it was negatively correlated with self-compassion (r = -0.590; p < 0.001) and mindful state (r = -0.383; p = 0.026). Moreover, we demonstrated that self-compassion mediates the relationship between emotional dysregulation and EE (ab product estimate = 0.043, 95% confidence interval [95%CI] 0.003-0.084).

**Conclusion:**

Our findings contribute to the literature by identifying psychological factors that could mediate the association between emotional dysregulation and EE, enabling identification of more effective eating behavior intervention targets for patients with GAD.

## Introduction

Emotional eating (EE) is characterized by eating in response to generally negative emotional stimuli.^1^ Studies have found a significant association between EE and high levels of symptoms of anxiety and depression. In anxious young people, EE may be a way of coping with hyper-arousal experiences, whereas in depression, EE may offer positive emotions.^2,3^ Dysregulation of negative emotions may be an important risk factor for EE,^4^ and experiencing negative emotions has been associated with subsequent overeating in some individuals.^5^

Presence of EE brings maladaptive behaviors and feeling of guilt besides being a significant risk factor for development of eating disorders such as binge eating disorder.^6,7^ A previous study reported that EE is more common in obese individuals than in normal weight individuals.^8^ Furthermore, EE was identified as an independent risk factor for weight gain in a 4-year follow-up of Korean twins.^9^ Moreover, EE is also associated with weight fluctuations, weight gain, and weight regain after treatment.^10-13^

Emotion regulation is characterized by the interaction between automatic and cognitive processes that influence the intensity, duration, and expression of emotions.^14^ Deficits in emotion regulation are often associated with employment of maladaptive strategies to regulate negative emotions, such as avoidance, rumination, self-harm, substance abuse, and/or eating.^14,15^ Facets of emotion dysregulation may contribute to development of EE^16^ and have been linked to weight gain.^17^ A meta-analysis of emotion regulation across different psychopathologies reported that individuals diagnosed with generalized anxiety disorder (GAD) presented deficits in emotion regulation related to emotional clarity, understanding, reactivity, and acceptance.^18^

GAD is a prevalent and disabling disorder^19^ and is associated with eating disorders^20^ and obesity.^21^ During the coronavirus disease 2019 (COVID-19) pandemic, it seems that anxiety rates in the general population may have been more than three times higher.^22^ The negative emotions experienced in GAD impair eating behavior, but there are very few studies evaluating the relationships between GAD, eating behavior, and body weight.

Mindfulness contributes to regulation of emotions and a more mindful state is associated with fewer symptoms of anxiety, depression, and binge eating disorder, among others.^23-27^ Self-compassion has been described as the non-judgmental acceptance of one’s own suffering, whilst also directing kindness towards one-self.^28,29^ Lower self-compassion scores are consistently associated with mental health symptoms such as anxiety, depression, narcissism, self-criticism, and avoidance.^29-33^ Preliminary evidence suggests that emotion regulation may be a mechanism of change in the relationship between self-compassion and mental health.^33-35^

There is an association between self-compassion and health behaviors such as healthy eating and physical activity.^36^ In addition, in a sample of university students, self-compassion had a negative correlation with consumption of sugar and fat (associated with isolation and over-identification).^37^ Brewer et al.^38^ suggest that self-compassion improves the relationship with food through the self-regulatory mechanism by moderating possible negative responses to failures and encouraging individuals to act from internal perceptions rather than extrinsic reward mechanisms.

Since GAD occurs twice as often in female patients^39^ and the risk for eating disorders is also higher in women,^40^ we aimed to investigate the relationship between emotional dysregulation and EE in women, evaluating potential mediators of this association. Based on the literature, we established an a priori hypothesis that self-compassion would mediate the relationship between emotional dysregulation (predictor) and EE as an outcome. Knowledge of these mechanisms may help to improve more effective strategies to deal with eating behavior in women with GAD.

## Methods

### Participants

The sample originated from a randomized clinical trial that evaluated the effectiveness of a mindfulness intervention in patients from the community diagnosed with GAD.^41^ Individuals were recruited to participate in this study through a local media advertisement. Potential participants were screened by telephone using the Generalized Anxiety Disorder 7-item Scale (GAD-7) and those who scored ≥ 10 on this scale were invited to go to the hospital to undergo an extensive clinical evaluation (assessed with the Mini International Neuropsychiatric Interview [MINI]) with a trained psychiatrist or psychologist.

Patients were included in the study if they were over 18 years old and had a primary diagnosis of GAD, according to the American Psychiatric Association Diagnostic and Statistical Manual of Mental Disorders, Fifth Edition (DSM-5), diagnostic criteria. Exclusion criteria were presence of eating disorders, bipolar disorder, psychotic disorders, substance use disorders (except tobacco), or suicidal ideation in the last 6 months. Patients could be recruited for the study if they fulfilled the diagnosis of major depression, providing it was not the primary diagnosis and depression symptom severity did not exceed 23 according to the Hamilton Depression Rating Scale (HAM-D). See the original paper for more details about the sample.^41^ This study was carried out in a hospital in Porto Alegre/Brazil, and was approved by the Ethics Committee at the Hospital de Clínicas de Porto Alegre (CAAE 61336416.0.0000.5327). All participants gave written informed consent before entering the study.

### Measures

#### Difficulties in Emotion Regulation Scale (DERS)

This instrument assesses levels of emotional dysregulation in six domains: non-acceptance of negative emotions; inability to engage in goal-driven behaviors when experiencing negative emotions; difficulty controlling impulsive behavior when experiencing negative emotions; limited access to emotional regulation strategies that are perceived as effective; lack of emotional awareness; and lack of emotional clarity. It contains 36 items scored on a 5-point Likert scale from 1 to 5.^14^ This scale has been validated for Portuguese from Portugal,^42^ and there is a version adapted for the Brazilian population.

#### Penn State Worry Questionnaire (PSWQ)

This is a self-rated scale designed to measure worry trait that has excellent internal consistency and good test-retest reliability.^43^ It comprises 16 Likert type items scored from 1 to 5 and has demonstrated good ability to discriminate individuals with GAD, not correlating with other measures of anxiety and worry.^43^ There is a validated version for Brazilians with adequate internal consistency.^44^

#### Action and Acceptance Questionnaire (AAQ)

This scale measures avoidance of internal experiences^45^ and is validated for Portuguese-Brazilian populations.^46^ It evaluates psychological flexibility, which is defined as the ability to contact more completely with the present moment, as a conscious human being, and to change or persist in behavior when to do so serves valued ends.^47^

#### Five Facet Mindfulness Questionnaire (FFMQ)

According to the authors of the FFMQ, mindfulness is a multifaceted construct with five distinct facets. This questionnaire consists of 39 self-report items that assess each individual’s tendency to be mindful in daily life. All items are answered on a Likert scale from 1 to 5. The five facets (subscales) of the original version achieved values indicating good internal consistency: observe = α 0.83; describe α 0.91; act consciously = α 0.87; not judging = α 0.87; and not reacting = α 0.75.^48^ There is a translated and validated version for Brazilian samples.^49^

#### Self-Compassion Scale (SCS)

The self-compassion scale was designed to measure self-compassion in three components: self-judgment versus self-kindness, sense of isolation versus common humanity, and hyper-identification versus mindfulness.^29^ Respondents score how they usually behave at difficult times on a scale comprising 26 5-point items. It has been translated and adapted to Brazilian Portuguese.^50^

#### The Three Factor Eating Questionnaire (TFEQ-R21)

This questionnaire is a self-administered instrument that evaluates cognitive restraint behaviors (six questions), EE (six questions), and uncontrolled eating (nine questions) and has good internal consistency.^1^ It contains 21 questions in which individuals rate statements as true or false on a four-point scale, where “1” is totally true and “4” is totally false. This instrument has been translated into Portuguese and validated for Brazilian women.^51^

#### Anthropometric evaluation

The anthropometric evaluation was based on body mass index (BMI) and body composition. Body weight was measured using a digital, calibrated scale, with capacity of 200kg (Toledo^®^, São Bernardo do Campo, Brazil). We measured height using a vertical millimeter stadiometer (HoltainLimited^®^, Crosswell, Wales, UK). Both were measured twice and we took the average of both measurements. We calculated BMI, defined as the weight in kilograms divided by the square of height in meters squared (kg/m^2^), to evaluate nutritional status. We used bioimpedance equipment (InBody230^®^, Perafita, Portugal) to assess body composition (percentage fat), following the requirements for bioimpedance assessment.

## Procedure

Female participants who met the criteria for a primary diagnosis of GAD signed an Informed Consent Form and answered the DERS, PSWQ, FFMQ, SCS, and AAQ questionnaires at baseline, before randomization. The anthropometric assessment was conducted afterwards.

## Statistical analysis

Data were expressed as mean and standard deviation (SD) for normally distributed continuous variables and as median and interquartile range (IQR) for non-normal distributed data. The distributions of variables were assessed using the Shapiro-Wilk test. We performed bivariate correlation analyses between all variables by estimating Pearson correlation coefficients. The SCS and FFMQ scales were reversed before the mediation analysis so that higher scores indicated lower levels of self-compassion and mindful state, facilitating interpretation.

Considering power of 80%, a 5% significance level,^52-54^ and a moderate correlation of r = 0.40 to 0.6,^55^ a sample of 30 individuals would be able to detect differences in the EE variable (TFEQ-EE and DERS), in accordance with previous studies that considered a correlation between EE and emotion regulation of 0.3 as clinically relevant.^56,57^

We conducted mediation analyses using bootstrapping techniques, a conditional modeling analysis that utilizes an ordinary least squares-based path analytical framework to test for both direct and indirect effects.^58^ The mediation model is a causal process in which X, the independent variable, affects Y, the dependent variable. Path c quantifies this effect, called the total effect of X on Y.^59^ Path a represents the causal effect of the independent variable on the proposed mediator, M. Path b represents the causal effect of the mediator on the dependent variable, controlling for the independent variable, whereas path c′ represents the causal effect of the independent variable on the dependent variable controlling for the mediator. In the language of causal analysis, c′ is the direct effect of X on Y and is distinguishable from the total effect, c, in that the direct effect partials out from the total effect that part of the causal effect that is shared with M. An indirect effect is the product of path a (the association between the predictor [x] and the proposed explanatory intermediary variable [m]) and path b (the association between the proposed intermediary variable [m] and the dependent variable [y], controlling for x). The indirect pathway is considered statistically significant if the 95% confidence interval (95%CI) around a*b does not include 0.^59^

All assumptions for the analysis were assessed and met (linear regression R^2^ = 0.926; autocorrelation p = 0.292; collinearity VIFscs = 3.02, VIFders = 1.04) and data were analyzed using Jamovi Computer Software (version 1.0). Tests were two-tailed with a significance level of < 0.05.

## Results

### Participants

The whole sample comprised 51 female patients diagnosed with GAD, but 17 were excluded because of missing data. In this study, we excluded individuals for not completely answering the following questionnaires: DERS (n = 15), the EE-TFEQ (n = 2), the SCS (n =17), the FFMQ (n = 16), the PSWQ (n = 16), and the AAQ (n = 13).

The median age of the 34 participants who completed all measures and questionnaires was 29 (IQR, 19 to 60) years and their socioeconomic data classified them at a medium socioeconomic level. Mean body fat percentage was high (36.9%) (SD = 8.69) and mean BMI was 27.9 (SD = 6.86), classified as overweight. The majority of our sample (65%) had excess weight. Table 1 shows the clinical and demographic characteristics of the sample.

**Table 1:** Descriptive statistics and Pearson’s coefficients for correlations between emotional dysregulation (Difficulties in Emotion Regulation Scale [DERS]), self-compassion (Self-Compassion Scale [SCS]), mindful state (Five Facet Mindfulness Questionnaire [FFMQ]), worry trait (Penn State Worry Questionnaire [PSWQ]), avoidance of internal experiences (Action and Acceptance Questionnaire [AAQ]), and emotional eating (Three Factor Eating Questionnaire [TFEQ])

	Mean	SD		1	2	3	4	5	6	7
1 Age	29^†^	19-60^†^		—						
2 Emotional eating	19^†^	11-24^†^	r	-0.182	—					
			p	0.302	—					
3 Emotional dysregulation	116	17.8	r	-0.390*	0.593***	—				
			p	0.023	< .001	—				
4 Self-compassion	2.4	0.6	r	0.404*	-0.590***	-0.511**	—			
			p	0.018	< .001	0.002	—			
5 Mindful state	110	20.1	r	0.491**	-0.383*	-0.358*	0.572***	—		
			p	0.003	0.026	0.037	< .001	—		
6 Worry trait	61.1	8.2	r	-0.419*	0.402*	0.541***	-0.469**	-0.411*	—	
			p	0.014	0.018	< .001	0.005	0.016	—	
7 Avoidance	33.2	7.9	r	-0.411*	0.565***	0.410*	-0.684***	-0.655***	0.486**	—
			p	0.016	< .001	0.016	< .001	< .001	0.004	—

SD = standard deviation.

* p < 0.05; ** p < 0.01; *** p < 0.001.

^†^ Median and interquartile range (IQR).

### Bivariate correlation analysis

EE was positively correlated with DERS (Pearson’s r = 0.593; p < 0.001; 95%CI 0.776-0.319), PSWQ (Pearson’s r = 0.402; p = 0.018; 95%CI 0.652-0.074), and AAQ (Pearson’s r = 0.565; p < 0.001; 95%CI 0.758-0.281); and negatively correlated with SCS (Pearson’s r = -0.590; p < 0.001; 95%CI -0.314 to -0.774) and FFMQ (Pearson’s r = -0.383; p = 0.026; 95%CI -0.051 to -0.638). BMI and percentage body fat were not correlated with any other variables (Table 1).

### Mediation analysis

**Figure 1:**
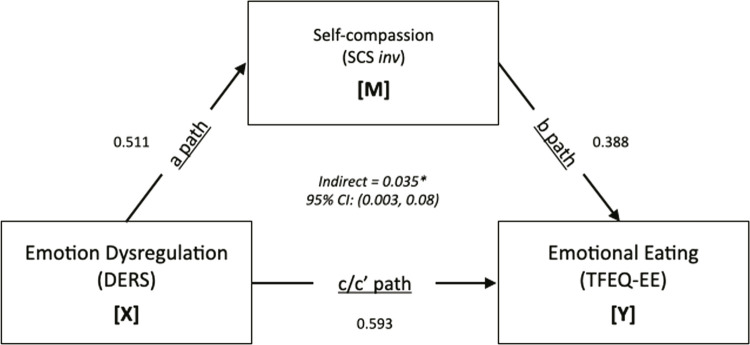
Conceptual model. A single path was conducted (X) on the outcome (Y). The Self-Compassion Scale (SCS) was reversed. 95%CI = 95% confidence interval; a path = effect of X on M; b path = effect of M on Y, controlling for X; c path = total effect of X on Y; c’ path = direct effect of X on Y controlling for M; DERS = Difficulties in Emotion Regulation Scale; EE = emotional eating; TFEQ = Three Factor Eating Questionnaire. * p < 0.05.

The mediation analysis for the relationship between DERS and EE only showed significant results when SCS *inv* was the mediator (Figure 1). All analyses conducted are described in the Supplementary Material S1 (online-only). Impairment in emotion dysregulation mediated 33.4% of the total effect of self-compassion impairment on EE impairment. The results indicated that higher reported levels of emotion dysregulation were associated with higher levels of EE, mediated by lower reported levels of self-compassion (a * b = 0.043, standard error [SE] = 0.02, 95%CI 0.003-0.084).

The total effects mediation model with emotion dysregulation predicting EE was significant (E = 0.131 SE = 0.0313 β = 0.593 t = 4.17 p < 0.001). The full model with self-compassion as mediator was significant (E = 2.4935, SE = 0.9841, β = 0.388, t = 2.53, p = 0.017). See Table 2 for more details.

**Table 2:** Indirect and total effects of emotion dysregulation on emotional eating via self-compassion

				95%CI*				
							
Type	Effect	Estimate	SE	Lower	Upper	β	z	p
Indirect	DERS ⇒ SCS_inv ⇒ EE	0.0436	0.02071	0.00304	0.0842	0.198	2.11	0.035
Component	DERS ⇒ SCS_inv	0.0175	0.00505	0.00760	0.0274	0.511	3.47	< .001
	SCS_inv ⇒ EE	2.4935	0.93970	0.65171	4.3353	0.388	2.65	0.008
Direct	DERS ⇒ EE	0.0870	0.03219	0.02387	0.1500	0.395	2.70	0.007
Total	DERS ⇒ EE	0.1306	0.03086	0.07011	0.1911	0.593	4.23	< .001

95%CI = 95% confidence interval; SE = standard error.

* Computed with method: Standard (delta method).

## Discussion

This study investigated some mediators of the relationship between emotional dysregulation and EE in women with GAD. Our data showed that EE was positively correlated with emotional dysregulation, worry trait, and avoidance of internal experiences, whereas it was negatively correlated with self-compassion and mindful state. Moreover, we demonstrated that self-compassion mediates the relationship between emotional dysregulation and EE.

EE is a dysfunctional coping strategy and a risk factor for various health conditions, including eating disorders.^60,61^ Anxiety seems to be associated with EE behaviors.^3,62^ Higher levels of EE were related to more severe neuroticism (e.g., anxiety and depression) in a treatment-seeking sample of adults with obesity.^63^ Moreover, EE is associated with emotion dysregulation^64^ and increased weight gain.^9,12,65,66^

Studies suggest that emotion dysregulation is independently related to eating in response to aversive events in adults with obesity.^64^ EE is considered a learned behavior^67^ and emotions become a trigger for eating impulsivity.^68^ Emotional eaters report greater consumption of sweet and high fat foods and more frequent snacking as compared to non-emotional eaters.^69,70^ Individuals with sweet craving had higher rates of uncontrolled eating and EE whereas anxiety symptoms are independently associated with sweet craving.^71^ This environment-reactive behavior is influenced by learning and experience^72^ and, in a similar way, some individuals with GAD paired pleasurable eating stimulus with a negative emotional experience. EE may regulate affect through release of dopamine after food consumption, which could increase positive affect, associated with subjective pleasure.^73,74^

Adults who endorsed the highest levels of EE were 13.38 times more likely to be overweight or obese than subjects who endorsed low levels of EE.^75^ EE is predictive of weight gain over time^12^ and of difficulty in losing weight.^76^ Jones et al.^4^ presented a mediation model in which higher levels of emotion dysregulation were associated with higher reported levels of EE, which in turn were related to higher BMI in adult smokers. This finding indicated that emotion dysregulation had an indirect effect on BMI mediated by EE.^4^ Mantau et al.^77^ investigated determinants of EE and showed that biological determinants (e.g., weight status) were less important than psychological (i.e., restrained eating) and situational (i.e., stress) factors in explaining food choice in response to a negative affective state.

In our sample, 65% of the women were overweight, and the level of self-compassion reported was lower than in the general population. The effect of emotion dysregulation on EE via lower self-compassion observed in our data is in agreement with the importance of psychological aspects and might represent a context in which higher levels of self-criticism and suffering could increase triggering of avoidance of negative feelings, driving eating to achieve prompt relief.

Elements of mindfulness and mindful eating are increasingly being incorporated into interventions designed to manage obesity-related eating behaviors.^78^ The skills that mindfulness fosters seem to increase self-regulation, improving awareness of emotions and sensations,^79-82^ which may be important for improving eating behaviors.^83^ However, we did not find a mediation effect via mindful state in our study.

According to Mantzios et al.,^84^ psychological interventions have identified that self-compassion may be the most relevant construct within mindfulness in terms of weight maintenance and loss. Meanwhile, Braun et al.^85^ propose that self-compassion can protect against dysfunctional eating behaviors through various mechanisms. The importance of developing self-compassion as a protective factor for emotional regulation in mental health and well-being is therefore highlighted.^31^

Self-compassion plays important roles in losing and maintaining weight,^86-89^ besides helping to develop more motivation and positive behaviors related to food.^32^ It also breaks the negative cycle of shame, dissatisfaction with body image, and the drive for thinness in women with and without eating disorders.^90^

Shame is a self-conscious emotion involving negative self-evaluation compounded by a desire to escape.^67,91,92^ Shame was strongly associated with unhealthy eating and with eating disorders.^67,93,94^ Liu et al.^95^ suggested that when shame was elicited in healthy women, they ate a higher number of snacks than women in whom shame was not elicited. Furthermore, individuals in an anxiety-with-shame group reported higher binge impulse than those in a group without shame.^67^ Self-compassion interventions aim to promote improvement in emotional regulation, facing negative emotions with self-kindness rather than negative self-appraisals (for example, self-criticism, shame).^15,96,97^

The main limitations of our study are its cross-sectional design and the small sample size. Although the sample size calculation showed that the study had the power to detect differences,^55-57^ our results must be interpreted with caution considering the multiple testing. Another possible limitation is assessment of participants with five different instruments totaling almost 150 items in a single assessment session, which could have influenced the reliability of the data collection process. Despite these limitations, our study has important strengths, including the a priori hypothesis tested with a robust analysis. We suggest more studies are needed to support our findings, but our data ensures future investigations on this theme.

These outcomes contribute to the literature by incorporating data on the functioning of EE in anxious patients and enriching possible alternative strategies for intervention in treatments for this condition. Further studies, especially longitudinal studies, are needed to indicate the best approach to improve EE in anxious patients.

## Conclusion

Findings highlight the influence of self-compassion as a mediator of the association between emotional dysregulation and EE in women with anxiety. Our findings could add to the literature by identifying psychological factors that are associated with EE, facilitating development of more effective interventions for this population with GAD and eating behavior dysfunction.

## References

[1] Stunkard AJ, Messick S (1985). The three-factor eating questionnaire to measure dietary restraint, disinhibition and hunger. J Psychosom Res.

[2] Eddy KT, Tanofsky-Kraff M, Thompson-Brenner H, Herzog DB, Brown TA, Ludwig DS (2007). Eating disorder pathology among overweight treatment-seeking youth: clinical correlates and cross-sectional risk modeling. Behav Res Ther.

[3] Goossens L, Braet C, Van Vlierberghe L, Mels S (2009). Loss of control over eating in overweight youngsters: the role of anxiety, depression and emotional eating. Eur Eat Disord Rev.

[4] Jones J, Kauffman BY, Rosenfield D, Smits JAJ, Zvolensky MJ (2019). Emotion dysregulation and body mass index: the explanatory role of emotional eating among adult smokers. Eat Behav.

[5] Sultson H, Kukk K, Akkermann K (2017). Positive and negative emotional eating have different associations with overeating and binge eating: construction and validation of the positive-negative emotional eating scale. Appetite.

[6] Bennett J, Greene G, Schwartz-Barcott D (2013). Perceptions of emotional eating behavior. A qualitative study of college students. Appetite.

[7] Haedt-Matt AA, Keel PK, Racine SE, Burt SA, Hu JY, Boker S (2014). Do emotional eating urges regulate affect? Concurrent and prospective associations and implications for risk models of binge eating. Int J Eat Disord.

[8] Patel KA, Schlundt DG (2001). Impact of moods and social context on eating behavior. Appetite.

[9] Sung J, Lee K, Song YM (2009). Relationship of eating behavior to long-term weight change and body mass index: the healthy twin study. Eat Weight Disord.

[10] Keller C, Siegrist M (2015). Ambivalence toward palatable food and emotional eating predict weight fluctuations. Results of a longitudinal study with four waves. Appetite.

[11] Torres SJ, Nowson CA (2007). Relationship between stress, eating behavior, and obesity. Nutrition.

[12] Koenders PG, van Strien T (2011). Emotional eating, rather than lifestyle behavior, drives weight gain in a prospective study in 1562 employees. J Occup Environ Med.

[13] Frayn M, Knäuper B (2018). Emotional eating and weight in adults: a review. Current Psychol.

[14] Gratz KL, Roemer L (2004). Multidimensional assessment of emotion regulation and dysregulation: development, factor structure, and initial validation of the difficulties in emotion regulation scale. J Psychopathol Behav Assess.

[15] Berking M, Whitley B, Berking M, Whitley B (2014). Affect regulation training.

[16] Ferrell EL, Watford TS, Braden A (2020). Emotion regulation difficulties and impaired working memory interact to predict boredom emotional eating. Appetite.

[17] Sainsbury K, Evans EH, Pedersen S, Marques MM, Teixeira PJ, Lähteenmäki L (2019). Attribution of weight regain to emotional reasons amongst European adults with overweight and obesity who regained weight following a weight loss attempt. Eat Weight Disord.

[18] Aldao A, Nolen-Hoeksema S, Schweizer S (2010). Emotion-regulation strategies across psychopathology: a meta-analytic review. Clin Psychol Rev.

[19] Wittchen HU (2002). Generalized anxiety disorder: prevalence, burden, and cost to society. Depress Anxiety.

[20] Dellava JE, Thornton LM, Hamer RM, Strober M, Plotnicov K, Klump KL (2010). Childhood anxiety associated with low BMI in women with anorexia nervosa. Behav Res Ther.

[21] Gariepy G, Nitka D, Schmitz N (2010). The association between obesity and anxiety disorders in the population: a systematic review and meta-analysis. Int J Obes.

[22] Santabárbara J, Lasheras I, Lipnicki DM, Bueno-Notivol J, Pérez-Moreno M, López-Antón R (2021). Prevalence of anxiety in the COVID-19 pandemic: an updated meta-analysis of community-based studies. Prog Neuropsychopharmacol Biol Psychiatry.

[23] Arch JJ, Craske MG (2006). Mechanisms of mindfulness: emotion regulation following a focused breathing induction. Behav Res Ther.

[24] Hill CL, Updegraff JA (2012). Mindfulness and its relationship to emotional regulation. Emotion.

[25] Grecucci A, Giorgetta C, Rattin A, Guerreschi C, Sanfey AG, Bonini N (2014). Time devours things: how impulsivity and time affect temporal decisions in pathological gamblers. PLoS One.

[26] Etkin A, Büchel C, Gross JJ (2015). The neural bases of emotion regulation. Nat Rev Neurosci.

[27] Guendelman S, Medeiros S, Rampes H (2017). Mindfulness and emotion regulation: insights from neurobiological, psychological, and clinical studies. Front Psychol.

[28] Gilbert P (2014). The origins and nature of compassion focused therapy. Br J Clin Psychol.

[29] Neff KD (2003). The development and validation of a scale to measure self-compassion. Self Identity.

[30] Leary MR, Tate EB, Adams CE, Allen AB, Hancock J (2007). Self-compassion and reactions to unpleasant self-relevant events: the implications of treating oneself kindly. J Pers Soc Psychol.

[31] MacBeth A, Gumley A (2012). Exploring compassion: a meta-analysis of the association between self-compassion and psychopathology. Clin Psychol Rev.

[32] Neff KD, Vonk R (2009). Self-compassion versus global self-esteem: two different ways of relating to oneself. J Pers.

[33] Inwood E, Ferrari M (2018). Mechanisms of change in the relationship between self-compassion, emotion regulation, and mental health: a systematic review. Appl Psychol Health Well Being.

[34] Terry ML, Leary MR, Mehta S, Henderson K (2013). Self-compassionate reactions to health threats. Pers Soc Psychol Bull.

[35] Diedrich A, Grant M, Hofmann SG, Hiller W, Berking M (2014). Self-compassion as an emotion regulation strategy in major depressive disorder. Behav Res Ther.

[36] Sirois FM, Kitner R, Hirsch JK (2015). Self-compassion, affect, and health-promoting behaviors. Health Psychol.

[37] Mantzios M, Egan H, Hussain M, Keyte R, Bahia H (2018). Mindfulness, self-compassion, and mindful eating in relation to fat and sugar consumption: an exploratory investigation. Eat Weight Disord.

[38] Brewer JA, Ruf A, Beccia AL, Essien GI, Finn LM, van Lutterveld R (2018). Can mindfulness address maladaptive eating behaviors? Why traditional diet plans fail and how new mechanistic insights may lead to novel interventions. Front Psychol.

[39] Steel Z, Marnane C, Iranpour C, Chey T, Jackson JW, Patel V (2014). The global prevalence of common mental disorders: a systematic review and meta-analysis 1980-2013. Int J Epidemiol.

[40] Striegel-Moore RH, Rosselli F, Perrin N, DeBar L, Wilson GT, May A (2009). Gender difference in the prevalence of eating disorder symptoms. Int J Eat Disord.

[41] Costa M, Gonçalves F, Tatton-Ramos T, Fonseca N, Schwinn J, Alves S (2021). A three-arm randomized clinical trial comparing the efficacy of a mindfulness-based intervention with an active comparison group and fluoxetine treatment for adults with generalized anxiety disorder. Psychother Psychosom.

[42] Coutinho J, Ribeiro E, Ferreirinha R, Dias P (2010). Versão portuguesa da Escala de Dificuldades de Regulação Emocional e sua relação com sintomas psicopatológicos. Rev Psiq Clin.

[43] Meyer TJ, Miller ML, Metzger RL, Borkovec TD (1990). Development and validation of the Penn State Worry Questionnaire. Behav Res Ther.

[44] Castillo C, Macrini L, Cheniaux E, Landeira-Fernandez J (2010). Psychometric properties and latent structure of the Portuguese version of the Penn State Worry Questionnaire. Span J Psychol.

[45] Hayes SC, Strosahl K, Wilson KG, Bissett RT, Pistorello J, Polusny MA (2004). Measuring experiential avoidance: a preliminary test of a working model. Psychol Rec.

[46] Barbosa LM, Murta SG (2015). Propriedades psicométricas iniciais do Acceptance and Action Questionnaire - II - versão brasileira. Psico-USF.

[47] Hayes SC, Luoma JB, Bond FW, Masuda A, Lillis J (2006). Acceptance and commitment therapy: model, processes and outcomes. Behav Res Ther.

[48] Baer RA, Smith GT, Hopkins J, Krietemeyer J, Toney L (2006). Using self-report assessment methods to explore facets of mindfulness. Assessment.

[49] Barros V (2014). Evidências de validade da versão brasileira do questionário das cinco facetas de mindfulness (FFMQ-BR). Psic Teor Pesq.

[50] Souza LKd, Hutz CS (2016). Adaptation of the Self-Compassion Scale for use in Brazil: evidences of construct validity. Temas Psicol.

[51] Natacci LC, Ferreira M (2011). The three factor eating questionnaire - R21: tradução para o português e aplicação em mulheres brasileiras. Rev Nutr.

[52] Agranonik M, Hirakata VN (2011). Cálculo de tamanho de amostra: proporções. Clin Biomed Res.

[53] Borges RB, Mancuso ACB, Camey AS, Leotti VB, Hirakata VN, Azambuja GS (2020). Power and sample size for health researchers: uma ferramenta para cálculo de tamanho amostral e poder do teste voltado a pesquisadores da área da saúde. Clin Biomed Res.

[54] Castro SMJ, Branco AC, Camey AS, Leotti VB, Hirakata VN, Borges RB (2021). PSS health: como calcular tamanho de amostra para estimar média, proporção e correlação. Clin Biomed Res.

[55] Dancey C, Reidy J (2006). Statistics without maths for psychology: using SPSS for Windows.

[56] Fisher NR, Mead BR, Lattimore P, Malinowski P (2017). Dispositional mindfulness and reward motivated eating: the role of emotion regulation and mental habit. Appetite.

[57] Willem C, Nandrino JL, Doba K, Roussel M, Triquet C, Verkindt H (2021). Interoceptive reliance as a major determinant of emotional eating in adult obesity. J Health Psychol.

[58] Hayes AF (2023). Introduction to mediation, moderation, and conditional process analysis.

[59] Preacher KJ, Hayes AF (2008). Asymptotic and resampling strategies for assessing and comparing indirect effects in multiple mediator models. Behav Res Methods.

[60] Eldredge KL, Agras WS (1996). Weight and shape overconcern and emotional eating in binge eating disorder. Int J Eat Disord.

[61] Farrow CV, Haycraft E, Blissett JM (2015). Teaching our children when to eat: how parental feeding practices inform the development of emotional eating--a longitudinal experimental design. Am J Clin Nutr.

[62] Nguyen-Rodriguez ST, Unger JB, Spruijt-Metz D (2009). Psychological determinants of emotional eating in adolescence. Eat Disord.

[63] Leehr EJ, Krohmer K, Schag K, Dresler T, Zipfel S, Giel KE (2015). Emotion regulation model in binge eating disorder and obesity--a systematic review. Neurosci Biobehav Rev.

[64] Gianini LM, White MA, Masheb RM (2013). Eating pathology, emotion regulation, and emotional overeating in obese adults with Binge Eating Disorder. Eat Behav.

[65] van Strien T, Herman CP, Anschutz DJ, Engels RC, de Weerth C (2012). Moderation of distress-induced eating by emotional eating scores. Appetite.

[66] van Strien T, Konttinen H, Homberg JR, Engels RC, Winkens LH (2016). Emotional eating as a mediator between depression and weight gain. Appetite.

[67] Wong M, Qian M (2016). The role of shame in emotional eating. Eat Behav.

[68] Jansen A, Nederkoorn C, van Baak L, Keirse C, Guerrieri R, Havermans R (2009). High-restrained eaters only overeat when they are also impulsive. Behav Res Ther.

[69] Camilleri GM, Méjean C, Kesse-Guyot E, Andreeva VA, Bellisle F, Hercberg S (2014). The associations between emotional eating and consumption of energy-dense snack foods are modified by sex and depressive symptomatology. J Nutr.

[70] O’Connor DB, Jones F, Conner M, McMillan B, Ferguson E (2008). Effects of daily hassles and eating style on eating behavior. Health Psychol.

[71] Penaforte FRO, Minelli MCS, Anastácio LR, Japur CC (2019). Anxiety symptoms and emotional eating are independently associated with sweet craving in young adults. Psychiatry Res.

[72] Gibson EL (2006). Emotional influences on food choice: sensory, physiological and psychological pathways. Physiol Behav.

[73] Haedt-Matt AA, Keel PK (2011). Revisiting the affect regulation model of binge eating: a meta-analysis of studies using ecological momentary assessment. Psychol Bull.

[74] Small DM, Jones-Gotman M, Dagher A (2003). Feeding-induced dopamine release in dorsal striatum correlates with meal pleasantness ratings in healthy human volunteers. Neuroimage.

[75] Ozier AD, Kendrick OW, Leeper JD, Knol LL, Perko M, Burnham J (2008). Overweight and obesity are associated with emotion- and stress-related eating as measured by the eating and appraisal due to emotions and stress questionnaire. J Am Diet Assoc.

[76] Braden A, Flatt SW, Boutelle KN, Strong D, Sherwood NE, Rock CL (2016). Emotional eating is associated with weight loss success among adults enrolled in a weight loss program. J Behav Med.

[77] Mantau A, Hattula S, Bornemann T (2018). Individual determinants of emotional eating: a simultaneous investigation. Appetite.

[78] Olson KL, Emery CF (2015). Mindfulness and weight loss: a systematic review. Psychosom Med.

[79] Guerrieri R, Nederkoorn C, Jansen A (2008). The interaction between impulsivity and a varied food environment: its influence on food intake and overweight. Int J Obes.

[80] Hall PA (2012). Executive control resources and frequency of fatty food consumption: findings from an age-stratified community sample. Health Psychol.

[81] Hemmingsson E (2014). A new model of the role of psychological and emotional distress in promoting obesity: conceptual review with implications for treatment and prevention. Obes Rev.

[82] Brace A, Yeomans MR (2016). The reinforcing value of palatable snack foods and its relationship to subtypes of behavioural and self-report impulsivity. Eat Behav.

[83] O’Reilly GA, Cook L, Spruijt-Metz D, Black DS (2014). Mindfulness-based interventions for obesity-related eating behaviours: a literature review. Obes Rev.

[84] Mantzios M, Egan H (2018). An exploratory examination of mindfulness, self-compassion, and mindful eating in relation to motivations to eat palatable foods and BMI. Health Psychol Rep.

[85] Braun TD, Park CL, Gorin A (2016). Self-compassion, body image, and disordered eating: a review of the literature. Body Image.

[86] Mantzios M, Wilson JC (2014). Making concrete construals mindful: a novel approach for developing mindfulness and self-compassion to assist weight loss. Psychol Health.

[87] Mantzios M, Giannou K (2014). Group vs. single mindfulness meditation: exploring avoidance, impulsivity, and weight management in two separate mindfulness meditation settings. Appl Psychol Health Well Being.

[88] Mantzios M, Wilson JC (2015). Mindfulness, eating behaviours, and obesity: a review and reflection on current findings. Curr Obes Rep.

[89] Hussein M, Egan H, Manztios M (2017). Mindful construal diaries: a less anxious, more mindful, and more self-compassionate method of eating [.

[90] Ferreira C, Pinto-Gouveia J, Duarte C (2013). Self-compassion in the face of shame and body image dissatisfaction: implications for eating disorders. Eat Behav.

[91] Lewis HB (1971). Shame and guilt in neurosis. Psychoanal Rev.

[92] Tangney JP (1996). Conceptual and methodological issues in the assessment of shame and guilt. Behav Res Ther.

[93] Hayaki J, Friedman MA, Brownell KD (2002). Emotional expression and body dissatisfaction. Int J Eat Disord.

[94] Sweetingham R, Waller G (2008). Childhood experiences of being bullied and teased in the eating disorders. Eur Eat Disord Rev.

[95] Liu (2015). Parenting style and disordered eating: the mediating role of shame.

[96] Gilbert P, Procter S (2006). Compassionate mind training for people with high shame andself-criticism: overview andpilot study of a group therapy approach. Clin Psychol Psychother.

[97] Neff KD, Germer CK (2013). A pilot study and randomized controlled trial of the mindful self-compassion program. J Clin Psychol.

