# Supplementary material for: Emotional eating in women with generalized anxiety disorder
Source: Trends Psychiatry Psychother. 2023 Sep 22;45:e20210399. doi: 10.47626/2237-6089-2021-0399 (PMC10597384; doi:10.47626/2237-6089-2021-0399)
Supplement: Supplementary file 1 [file 2238-0019-trends-45-e20210399-suppl01.pdf]

http://doi.org/10.47626/2237-6089-2021-0399

Supplementary Material S1

SCS Regression and Mediations

Regressions Results

Total effects predicting: TFEQ_EE

Names	Effect	Estimate	SE	Lower	Upper	β	df	t	p
DERS	DERS	0.131	0.0313	0.0668	0.194	0.593	32	4.17	< .001

TFEQ_EE = Three Factor Eating Questionnaire – Emotional Eating; DERS = Difficulties in Emotion Regulation Scale.

Mediator Model

Dependent variable: SCS_inv

Names	Effect	Estimate	SE	Lower	Upper	β	df	t	p
DERS	DERS	0.0175	0.00521	0.00690	0.0281	0.511	32	3.36	0.002

SCS_inv = Self-Compassion Scale; Inv = The scale was used inversely to measure self-compassion in a negative way, that is, the less self-compassion one had; DERS = Difficulties in Emotion Regulation Scale.

Full model predicting TFEQ_EE

Names	Effect	Estimate	SE	Lower	Upper	β	df	t	p
SCS_inv	SCS_inv	2.4935	0.9841	0.4864	4.501	0.388	31	2.53	0.017
DERS	DERS	0.0870	0.0337	0.0182	0.156	0.395	31	2.58	0.015

TFEQ_EE = Three Factor Eating Questionnaire – Emotional Eating; SCS_inv = Self-Compassion Scale; Inv = The scale was used inversely to measure self-compassion in a negative way, that is, the less self-compassion one had; DERS = Difficulties in Emotion Regulation Scale.

http://doi.org/10.47626/2237-6089-2021-0399

Mediation Estimates (SCS)

				95% Confidence Interval			
**Effect**	**Label**	**Estimate**	**SE**	**Lower**	**Upper**	**Z**	**p**
Indirect	a × b	0.0436	0.0222	0.00623	0.0954	1.97	0.049
Direct	c	0.0870	0.0285	0.03081	0.1470	3.05	0.002
Total	c + a × b	0.1306	0.0251	0.08378	0.1813	5.20	< .001

Path Estimates

						95% Confidence Interval			
			**Label**	**Estimate**	**SE**	**Lower**	**Upper**	**Z**	**p**
DERS	→	SCS_inv	a	0.0175	0.00495	0.00861	0.0279	3.54	< .001
SCS_inv	→	TFEQ_EE	b	2.4935	1.04836	0.37433	4.4497	2.38	0.017
DERS	→	TFEQ_EE	c	0.0870	0.02853	0.03081	0.1470	3.05	0.002

Mediation Estimates (AAQ)

				95% Confidence Interval			
**Effect**	**Label**	**Estimate**	**SE**	**Lower**	**Upper**	**Z**	**p**
Indirect	a × b	0.0349	0.0205	0.00249	0.0820	1.71	0.088
Direct	c	0.0957	0.0243	0.04600	0.1426	3.94	< .001
Total	c + a × b	0.1306	0.0233	0.08401	0.1757	5.61	< .001

Path Estimates

						95% Confidence Interval			
			**Label**	**Estimate**	**SE**	**Lower**	**Upper**	**Z**	**p**
DERS	→	AAQ	a	0.1820	0.0739	0.0403	0.338	2.46	0.014
AAQ	→	TFEQ_EE	b	0.1919	0.0711	0.0397	0.318	2.70	0.007
DERS	→	TFEQ_EE	c	0.0957	0.0243	0.0460	0.143	3.94	< .001

SCS = Self-Compassion Scale; DERS = Difficulties in Emotion Regulation Scale; SCS_inv = Self-Compassion Scale; Inv = The scale was used inversely to measure self-compassion in a negative way, that is, the less self-compassion one had; TFEQ_EE = Three Factor Eating Questionnaire – emotional; AAQ = Action and Acceptance Questionnaire.

http://doi.org/10.47626/2237-6089-2021-0399

Mediation Estimates (FFMQ)

				95% Confidence Interval			
**Effect**	**Label**	**Estimate**	**SE**	**Lower**	**Upper**	**Z**	**p**
Indirect	a × b	0.0154	0.0148	-0.0121	0.0494	1.04	0.299
Direct	c	0.1152	0.0283	0.0597	0.1707	4.06	< .001
Total	c + a × b	0.1306	0.0242	0.0829	0.1765	5.40	< .001

Path Estimates

						95% Confidence Interval			
			**Label**	**Estimate**	**SE**	**Lower**	**Upper**	**Z**	**p**
DERS	→	FFMQ - inv	a	0.4048	0.1781	0.0802	0.775	2.27	0.023
FFMQ - inv	→	TFEQ_EE	b	0.0380	0.0329	-0.0260	0.107	1.15	0.248
DERS	→	TFEQ_EE	c	0.1152	0.0283	0.0597	0.171	4.06	< .001

Mediation Estimates (PSWQ)

				95% Confidence Interval			
**Effect**	**Label**	**Estimate**	**SE**	**Lower**	**Upper**	**Z**	**p**
Indirect	a × b	0.0137	0.0245	-0.0308	0.0663	0.558	0.577
Direct	c	0.1169	0.0338	0.0501	0.1820	3.455	< .001
Total	c + a × b	0.1306	0.0244	0.0796	0.1764	5.348	< .001

Path Estimates

						95% Confidence Interval			
			**Label**	**Estimate**	**SE**	**Lower**	**Upper**	**Z**	**p**
DERS	→	PSWQ	a	0.2484	0.0722	0.1101	0.391	3.443	< .001
PSWQ	→	TFEQ_EE	b	0.0550	0.0903	-0.1276	0.225	0.609	0.543
DERS	→	TFEQ_EE	c	0.1169	0.0338	0.0501	0.182	3.455	< .001

FFMQ = Five Facet Mindfulness Questionnaire; DERS = Difficulties in Emotion Regulation Scale; Inv = The scale was used inversely to measure self-compassion in a negative way, that is, the less self-compassion one had; TFEQ_EE = Three Factor Eating Questionnaire – Emotional Eating; PSWQ = Penn State Worry Questionnaire.
